# Benchmarking short-, long- and hybrid-read assemblers for metagenome sequencing of complex microbial communities

**DOI:** 10.1099/mic.0.001469

**Published:** 2024-06-25

**Authors:** Gleb Goussarov, Mohamed Mysara, Ilse Cleenwerck, Jürgen Claesen, Natalie Leys, Peter Vandamme, Rob Van Houdt

**Affiliations:** 1 Microbiology Unit, Belgian Nuclear Research Centre (SCK CEN), Mol, Belgium; 2 Laboratory of Microbiology and BCCM/LMG Bacteria Collection, Faculty of Sciences, Ghent University, Ghent, Belgium; 3 Bioinformatics group, Information Technology & Computer Science, Nile University, Giza, Egypt

**Keywords:** assembly, DNA mock metagenome, long-read sequencing, sequencer benchmark, software benchmark

## Abstract

Metagenome community analyses, driven by the continued development in sequencing technology, is rapidly providing insights in many aspects of microbiology and becoming a cornerstone tool. Illumina, Oxford Nanopore Technologies (ONT) and Pacific Biosciences (PacBio) are the leading technologies, each with their own advantages and drawbacks. Illumina provides accurate reads at a low cost, but their length is too short to close bacterial genomes. Long reads overcome this limitation, but these technologies produce reads with lower accuracy (ONT) or with lower throughput (PacBio high-fidelity reads). In a critical first analysis step, reads are assembled to reconstruct genomes or individual genes within the community. However, to date, the performance of existing assemblers has never been challenged with a complex mock metagenome. Here, we evaluate the performance of current assemblers that use short, long or both read types on a complex mock metagenome consisting of 227 bacterial strains with varying degrees of relatedness. We show that many of the current assemblers are not suited to handle such a complex metagenome. In addition, hybrid assemblies do not fulfil their potential. We conclude that ONT reads assembled with CANU and Illumina reads assembled with SPAdes offer the best value for reconstructing genomes and individual genes of complex metagenomes, respectively.

**Impact Statement**

Microbiomes play a profound role in both environmental health and human well-being, and understanding them is becoming an important research field. Each of the leading sequencing technologies (generating short or long DNA reads) has its own advantages and drawbacks. Therefore, hurdles still need to be taken to enhance the output of metagenomics (i.e. the study of genetic material from a whole community of organisms). In this study we compared the efficacy of different assemblers that use short, long or both read types on a complex community that was created by combining 227 bacterial strains with varying degrees of relatedness. It became clear that their performance varied widely and many of them are not suited to handle such a complex metagenome. These insights are useful for evaluating and intepreting metagenomics results as well as for guiding the selection of an appropriate technology and tool.

## Data Summary

The authors confirm all supporting data, code and protocols have been provided within the article or through supplementary data files.

## Introduction

Bacteria have colonized almost all ecosystems on Earth [1], where they act as free-living organisms that process organic and inorganic compounds [2], as symbionts that are necessary for the proper functioning of larger organisms [3], or as pathogens. Moreover, their relative simplicity facilitates exploiting their potential in manufacturing chemicals and biotechnical applications, and may hold the key to understanding the origin of life. However, only a small fraction of bacterial diversity has been properly characterized with only around 20 000 validly named species [4] out of an estimated 10^6^ to 10^12^ [5,6]. Although isolation and cultivation are the golden standard for bacterial characterization, it requires considerable efforts and many cannot be cultivated using standardized approaches [7,8]. Furthermore, not all environments are studied to the same extent, unravelling only a fraction of their full potential. Metagenomics can help to fill these knowledge gaps as it enables genome, gene and functional analysis without the need for a cultivation step.

However, metagenomics still faces hurdles as genomes are sequenced in fragments, which must then be separated according to their source organism and assembled into full genomes using specialized software [9]. At present, there are two types of competing sequencing technologies: paired short-read sequencing (dominated by Illumina), and long-read sequencing provided by Pacific Bioscience (PacBio) and Oxford Nanopore Technologies (ONT). Short-read sequencing is currently the dominant approach because of its low cost, low error rate and the fact that it has been on the market longer. Its primary drawback is the limited length of the reads, at only 100–300 bases per read, which results in fragmented assemblies because genomes often contain a large number of identical sequences (known as repeats) that can be thousands of nucleotides long. Long-read sequencing, such as ONT and PacBio, focuses on producing the longest possible reads, often above 50 kb. Originally, this increase in length also resulted in a lower accuracy, but both ONT and PacBio have been continually improving their methods. Recently, PacBio introduced the circular consensus sequencing (CCS) mode on its Sequel system. The latter generates high-fidelity (HiFi) consensus sequences, mostly 4–24 kb in length, from repeatedly produced subreads from a single circularized template [10]. In a critical step, sequencing reads need to be assembled and many tools have been developed to assemble short [11], long [12] and hybrid, i.e. short and long, reads [13,14]. However, many of these were designed for genomics and not metagenomics, urging a careful evaluation of assembled genomes in order to guarantee their quality and avoid misleading conclusions [15,16].

A limited number of studies have evaluated different metagenomics approaches based on either defined mock metagenomes [12,17], synthetic communities [12,18,20] or real metagenomes [21,22]. For instance, analysis of two mocks, one with 11 strains (ZymoBIOMICS), and one with 10 bacterial strains and two yeast strains [17] showed that CANU, Flye and Raven were the best out of a set of 10 assemblers [12]. While the evaluation of two other mocks, containing 20 bacterial strains each, showed that metaflye, CANU, OPERA-MS and WTDBG did not significantly outperform each other [23]. The use of synthetic communities, simulating complex mock metagenome sequenced by short reads, showed that MEGAHIT performed generally better than the Merega, Ray, Minia and Velour assemblers [24,25]. Evaluation of assemblers with real metagenomes showed that SPAdes [21], CANU and Flye [22] performed well.

Each approach to evaluate assemblers has its own drawbacks. The primary drawback of mock communities is the difficulty in creating and maintaining them, which is why they usually contain only a few genomes. Synthetic communities based on simulated reads may not be entirely representative of real sequencing results in terms of error profiles and reported quality scores, as well as read lengths in the case of long-read sequencing [26]. Furthermore, some short-read assemblers, including MEGAHIT, performed significantly better on simulated data, likely because of the more uneven coverage of real data [20,27]. Using real metagenomes limits the number of useful evaluation criteria in addition to possible discrepancies between the used reference genomes and studied metagenome [21,22].

In the present study, we evaluated commonly used assemblers for *de novo* metagenome assembly of a highly diverse mock metagenome sequenced with short-read Illumina, and long-read ONT and HiFi. By combining genomic DNA of 227 individual strains with varying degrees of relatedness, we overcame the primary drawback of the current mocks and revealed the limits of metagenome sequencing. In addition, by subsampling, we also provided indications for metagenome sequencing depth, thereby optimizing cost–benefit.

## Methods

### Data collection

As detailed previously [28,29], the input material for the benchmarking dataset was generated by pooling even amounts (by mass) of genomic DNA from 227 bacterial strains belonging to eight phyla (Actinobacteria, Bacteroidetes, Deinococcus-Thermus, Firmicutes, Fusobacteria, Planctomycetes, Proteobacteria and Verrucomicrobia), 19 classes, 47 orders, 85 families, 175 genera and 197 species, designated as the complex mock community, HC227 (Table S1). The genome of each strain was previously sequenced individually [29] and the final assemblies for these reference genomes totalled 1.002 Gb. Next, the HC227 mock was sequenced on Illumina Novaseq [2×150 paired-end (PE); Baseclear, Leiden, The Netherlands], PacBio Sequel (University of Antwerp, Belgium) and Nanopore PromethION (Nottingham University, UK). For the latter, a native-DNA sequencing library was prepared using the Genomic DNA by Ligation PromethION Kit (Oxford Nanopore Technologies; SQK-LSK109). The library was run on the PromethION platform on an R9.4.1 PromethION flow cell (Oxford Nanopore Technologies; FLO-PRO002) and called using the Guppy 3.2.6 basecaller.

### Data preparation

**Fig. 1.**
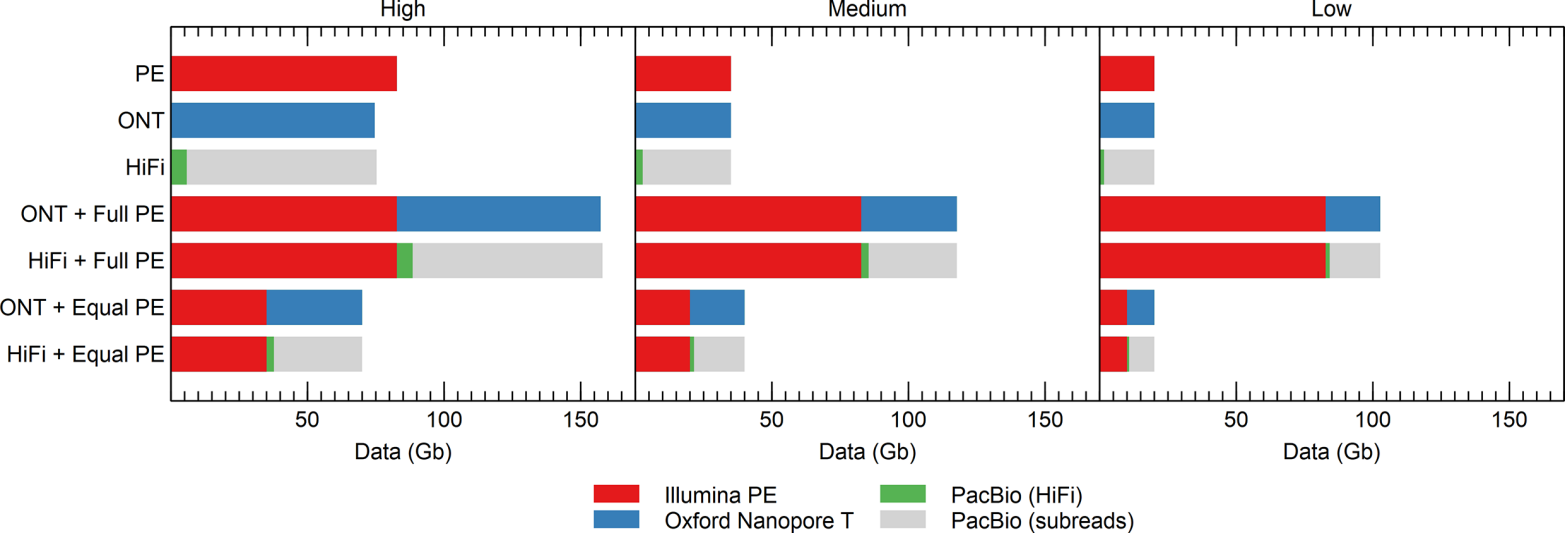
Size of the datasets used in this study, color-coded according to the sequencing technology. PacBio data is shown in two colours because it was subsampled according to subread (grey) coverage, but HiFi reads (green) were used in further analyses.

In addition to the complete read datasets, random subsampling was performed to 35, 20 and 10 Gb for each sequencing output. For Illumina PE and ONT reads, this was done with Rasusa [30]. PacBio HiFi reads were subsampled with a specifically written python script, as there is no one-to-one relation between the size of the HiFi data and the subread coverage. Briefly, the length of the subreads were first calculated and then summed to infer the polymerase read length [i.e. the total number of bases produced from a ZMW (zero-mode waveguide) after trimming the low-quality regions]. A number of polymerase reads (which are defined as a sequence of nucleotides incorporated by the DNA polymerase while a circular SMRTbell template) were then selected to attain the desired sequencing depth, and all the HiFi reads that matched the selected polymerase reads were used for further analysis (Fig. S1). For single-technology assembly, the datasets were subsampled at different depths. For hybrid assembly, short- and long-read datasets with equal coverage were combined (e.g. 35 Gb Illumina PE and 35 Gb ONT) in addition to the full Illumina dataset being combined with either ONT or HiFi reads subsampled at different depths (e.g. 82.7 Gb Illumina PE and 35 Gb ONT). All datasets used are shown in Fig. 1, depending on the fraction of reads used, these datasets were labelled as having ‘high’, ‘medium’ or ‘low’ coverage.

### Assembly and polishing of read datasets

Illumina PE reads were assembled with SPAdes v3.14 (--meta flag) [31] and megahit v1.2.9 (meta-sensitive preset) [32]. ONT reads were assembled with Shasta v0.7.0 (--consensusCaller Bayesian:guppy-3.4.4-a) [33], WTDBG v2.5 (-x ont, -g 1 g) [34], Minipolish v0.1.3 [26], canu v2.1.1 (genomeSize=1 g, minInputCoverage=3) [35] and Flye v2.8.2 (--meta flag) [36]. HiFi reads were assembled with wtdbg v2.5 (-x sequel, -g 1 g) [34], Minipolish v0.1.3 [26], canu v2.1.1 (genomeSize=1 g, minInputCoverage=1, stopOnLowCoverage=0) [35] and Flye v2.8.2 (--meta flag) [36]. Hybrid assembly was performed with SPAdes (--meta flag), opera-ms v0.9.0 (polishing disabled) [14], haslr v0.8a1 [13] and Unicycler v0.4.9b [37]. In addition, hybrid assembly was also done by performing long-read assembly with canu and polishing the resulting contigs with Illumina PE reads using Pilon v1.24 [38], freebayes v1.3.4 [39] and a combination of Bowtie2 v2.3.4.1 [40] and bcftools v1.11 [41]. For all programmes, genome size was set to 1 Gb (if to be specified), technology-specific presets were selected (if available) and default parameters were used unless specified.

### Assessing read and assembly quality

To evaluate read quality, reads were aligned to the combined reference genomes using Minimap2 v2.17-r941 (--secondary=no) [42]. Coverage and error rate associated with each genome were determined using nucops samcov (https://github.com/GlebGoussarov/nucops), with coverage calculated as the [number of mapped bases] / [genome length] and error rate as the [number non-matching bases (single-base substitutions)]/[total number of mapped bases].

The assembly quality was evaluated using MetaQUAST v5.1.0rc1 [43]. In addition to the default parameters, we used the –fragmented and the --unique_mapping flag to avoid overestimating misassemblies, and the recovered genome fractions and associated error rates, respectively. Completeness, fragmentation, indel rates, mismatch rates and inter-reference misassemblies [i.e. misassembly events (breakpoints) where the flanking sequences align on different reference genomes] were collected for each reference and also averaged to produce a single value. Fragmentation was computed by dividing the number of contigs by the genome fraction.

In addition to the statistics provided by MetaQUAST, we also evaluated the relative performance of assemblers. This was done with two metrics: the total number of genes from the original genome that could be identified (Prodigal v2.6.3) [44] and average nucleotide identity (ANI) computed with FastANI (v1.33) [45]. In both cases, a higher value indicated that an assembler more closely managed to reconstruct the original genome.

### Computing power

For this study, we had access to two systems running CentOS-7 and CentOS-8, respectively. The CentOS-7 system used the Intel(R) Xeon(R) E5-2690 v4 CPU, running at 2.60 GHz with 56 cores and 251 GB of RAM, whereas the CentOS-8 system used the Intel(R) Xeon(R) Gold 6254 CPU, running at 3.10 GHz with 72 cores and 187 GB of RAM. Analyses were split across the two machines according to availability and memory requirements.

## Results

### Raw read quality and error rates

Our HC227 mock DNA was sequenced with high-coverage Illumina 2×150 PE reads, and ONT and PacBio long reads, yielding 82.7, 74.6 and 75.3 Gb data, respectively (Table 1). For PacBio, this number corresponds to the initial size of the subreads, prior to being consolidated into 5.83 Gb of HiFi reads.

**Table 1.** Summary of sequencing data

Method	Platform	Total cost (€)*	Size (Gb)	Cost per Gb (€)	Reads (M)	Read length (bp)†	
						P1	P99
PE	Illumina‡	2515	82.70	30.41	572	58	151
ONT	PromethION	2924	74.60	39.19	13.70	248	43 231
HiFi§	PacBio sequel	5808	5.83	997.90	0.88	2039	15 619
			(75.3)	(77.13)	(14.60)	(70)	(14387)

*Cost is given as an indication as it is based on specific negotiations.

†P: percentile.

‡Novaseq.

§For HiFi reads, the sub-read output is mentioned between brackets.

We observed overall error rates of 10.5, 2.15 and 0.31 % for ONT, HiFi and Illumina PE, respectively. For ONT and HiFi reads, these were comparable with previous studies, being 11.2 % [46] and 1.72 % [47], respectively. As we did not filter PacBio HiFi reads by length, this could account for the slightly higher error rate compared to [47]. As the median error rate of Novaseq-produced Illumina PE reads is 0.1±0.35 % [48], our results were within the expected range. We found a clear relation between the error rate and the genome’s GC content for Illumina PE and PacBio HiFi reads (Fig. S2), which has also been reported by other studies [49,50].

On average, the Illumina PE, ONT and HiFi reads covered 99.1, 99.7 and 93.2 % of each genome in the HC227 mock, respectively. These genome fractions are the theoretical maximum a *de novo* assembly could achieve from these read sets and can be used to evaluate the ability of assemblers to retain available information. In addition, we found that GC-rich genomes appeared to be overrepresented in Illumina PE reads, which has been observed previously [51]. Contrary, no bias was observed for ONT and PacBio HiFi reads, suggesting that the latter are more suitable for estimating relative species abundance (Fig. S3).

### Assemblers evaluated

Our main goal was to evaluate the performance of different sequencing platforms and assemblers for accurately reconstructing complex metagenomes. To this end, we compared a variety of state-of-the-art *de novo* assemblers that can handle Illumina PE, ONT or PacBio reads on a complex mock community. Reference-based assemblers were not included, as we specifically focused on using metagenomics as a tool to explore environments, in which a large number of poorly studied or novel strains could be present. Concretely, reconstruction of genomes was assessed first, which required the lowest fragmentation, highest completeness and most minimal inter-reference misassemblies (chimaeras) possible. Second, studying genes was assessed, for which achieving the lowest error rate was of primary importance.

For Illumina PE, we tested SPAdes and megahit, which are the most prominent and best-performing short-read assemblers [11,20, 21, 24]. For ONT and HiFi reads, we tested Shasta, wtdbg, Minipolish, canu and Flye. Only canu and Flye were retained for comparison, as Shasta, wtdbg and Minipolish failed to assemble at least one of our datasets (Table S2). For combinations of short (Illumina PE) and long (ONT or HiFi) reads, SPAdes, haslr, MaSuRCA, opera-ms and Unicycler were tested. With the exception of haslr, which has not yet been assessed in other studies, all are standalone tools that were shown to perform well [11,12, 26]. Nevertheless, only SPAdes, which is designed to handle hybrid and metagenomic reads (though unable to handle long reads solely), and opera-ms were retained for comparison. HASLR and Unicycler, which are designed for assembling individual genomes, were unable to handle the full datasets with the available memory (252 GB of RAM). MaSuRCA (v4.0.1 and v4.0.4) produced a persistent error, which stated that the ‘input data should be checked’ without further clarifications. In addition, we also included long-read assemblers followed by a polishing step with Illumina PE reads. However, Pilon could not be run on our data due to RAM constraints, and freebayes systematically performed worse than our bowtie2 bcftools implementation (Table S3), revealing their shortcomings for complex metagenomes.

### Performance of single-technology assemblies

**Fig. 2.**
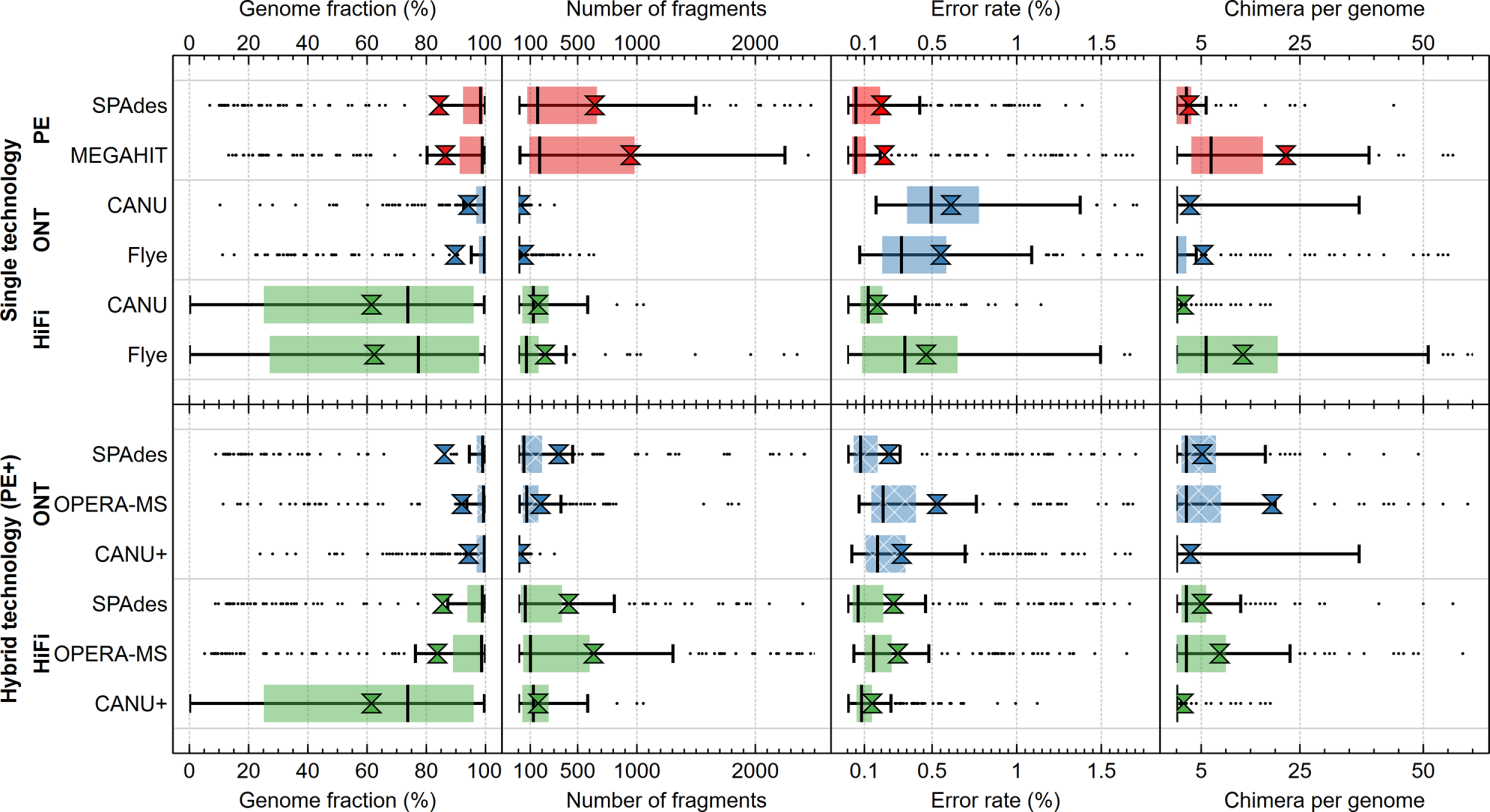
Box plots with the 1.5 IQR (interquartile range) of genome fraction, fragmentation, error rate and inter-reference misassemblies (chimaeras) collected for the different assemblers on the single (top panel) and hybrid (bottom panel) technology datasets. Assemblers were evaluated according to four criteria: genome fraction (i.e. the fraction of the reference genome that was found it the assembled metagenome), genome fragmentation (i.e. the number of fragments, computed by dividing the number of contigs by the genome fraction), error rate (i.e. the sum of the number of mismatches and the length of all short indels), and the number of inter-reference misassemblies or chimaeras (i.e. the number of contigs that partially mapped to another genome). The size of the input dataset is shown on the right. Hybrid assemblies were performed with either full Illumina PE reads and subsampled long reads (b, top panel) and equally subsampled short and long reads (b, bottom panel).

**Fig. 3.**
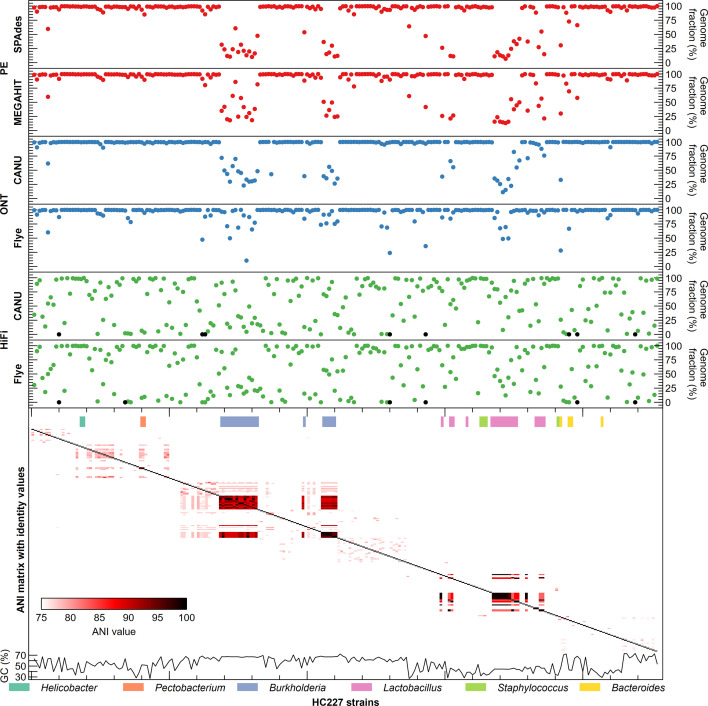
Genome fractions of the different strains in HC227 assembled by SPAdes and megahit starting from Illumina PE reads (red), and canu and Flye starting from ONT (blue) and HiFi (green) reads (black represents genomes that were not assembled). Genomes are ranked based on the ANI matrix with identity values, genomes from the same genus are color-coded (above ANI matrix). GC content of the genomes is shown below the ANI matrix.

The performance of the individual sequencing technologies and assemblers on the complex HC227 mock metagenome at full sequencing depth (roughly around 77 Gb) was assessed first. The average genome fraction covered by the assembly of Illumina PE reads was 86.4 and 84.3 % for megahit and SPAdes, respectively. This was considerably less than the best value for ONT reads, where a genome fraction of 94.2 % was achieved with canu. Noteworthy, although the PacBio subreads contained on average 93.2 % of a give genome, neither Flye nor canu recovered more than 63 % (Figs 2 and S1–S7, Table S3). As expected, considering the short-read length, Illumina PE assemblies were significantly more fragmented than the ONT and PacBio HiFi assemblies (Figs 2 and S4–S7, Table S3). Assembly of genomes from closely related strains was more complete for the ONT assemblies, followed by the Illumina PE and PacBio HiFi assemblies (Figs3^ 4^ and S8). All genomes were (partly) assembled only for the ONT and Illumina PE datasets. However, the effect was not only noticeable for closely related strains based on ANI (i.e. taxonomy), but also those with similar GC content (Figs3^ 4^ and S8). In fact, the genomes that were not assembled from the PacBio HiFi dataset have a similar GC content but belong to different species/genera (Figs3^ 4^ and S8).

**Fig. 4.**
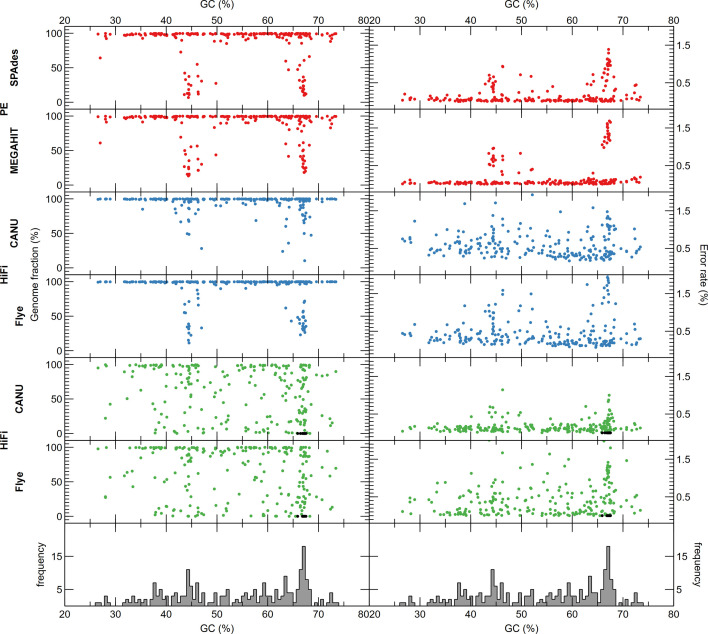
Genome fractions (left panel) and error rate (right panel) of the different strains in HC227 assembled by SPAdes and megahit starting from Illumina PE reads (red), and canu and Flye from ONT (blue) and HiFi (green) reads (black represents genomes that were not assembled). Genomes are ranked based on GC content. Bottom panel shows histogram of GC content for the HC227 genomes (100 bins).

In terms of error rate, Illumina PE reads assembled with SPAdes (0.20 %) or megahit (0.22 %) outperformed ONT reads assembled with canu (0.61 %) and Flye (0.55 %), as well as PacBio HiFi reads assembled with Flye (0.47 %), but not PacBio HiFi reads assembled with canu (0.18 %) (Figs 2 and S4–S7, Table S3). Finally, the lowest number of chimeric contigs per genome was observed for PacBio HiFi assemblies, but it should be noted that these also had the lowest genome fraction, reducing the number of expected chimeric contigs. Generally, the number of chimeric contigs depended primarily on the assembler rather than the technology.

For the Illumina PE reads, megahit produced a more complete assembly than SPAdes (86.4 % versus 84.3 %), but this was considerably more fragmented (946.6 versus 644.6) and contained more chimeric contigs per genome (22.15 versus 2.63). As indicated, the error rate of the two assemblers was roughly the same (0.22 % for megahit and 0.20 % for SPAdes), but megahit had fewer indels and more mismatches (Table S3). We conclude that both Illumina PE assemblers have their value, but based on fragmentation and the number of chimeric contigs, SPAdes is preferred over megahit for most applications. Our results are in line with other studies on metagenomes, which showed that SPAdes outperformed megahit [20,21, 52].

For long reads, ONT assemblies had a lower fragmentation and a higher average genome fraction than PacBio HiFi assemblies, making them more suitable for obtaining strain-resolved genomes from complex microbial communities. Although both long-read technologies had not yet been compared for sequencing a complex mock metagenome, sequencing low-complexity whey starter cultures did show that ONT allowed better repeat resolution than PacBio reads [53]. However, PacBio HiFi assembly could be considered for applications where high error rates are problematic, e.g. when looking at single nucleotide polymorphisms. Although both Flye and canu performed well for ONT- and PacBio-based genome assemblies [13], some studies tended to favour Flye [26,54]. Nevertheless, Flye’s required RAM precluded it being usable for large genomes such as the human genome [13]. For metagenomes, Flye was the best suited assembler of ONT reads from two different commercially available mock communities [12], whereas canu performed better on more complex metagenomes sampled from wastewater treatment plants [22]. These studies also indicated higher error rates for ONT-based assemblies. Noteworthy, our conclusions are based on similar sequencing depths of ONT and PacBio subreads, as achieving comparable depth in terms of HiFi reads would have required a significantly higher investment. This difference in cost between ONT and PacBio, also other favoured ONT sequencing as a basis for comparing assemblers in other studies [55].

As a result, we conclude that Illumina PE sequencing currently remains superior to both long-read technologies. This is especially true for the analysis of genes, as it provides a significantly lower error rate than ONT reads and enables the complete sequencing of complex samples at reasonable sequencing depths, contrary to PacBio. If high completeness and low fragmentation need to be achieved without error rate being critical, ONT sequencing is preferred. Noteworthy, with the more recently released R10.4 chemistry, ONT read accuracy is comparable to that of Illumina reads [56], which would probably favour ONT sequencing.

### Performance of hybrid assemblies

Next, we evaluated the benefit of using hybrid assemblies over single-technology assemblies. On the one hand, if the correct sequences can be properly identified, one would expect that more information would result in better quality. On the other hand, if data from different sequencing methods are interpreted inconsistently, hybrid assemblers might perform worse than their single-technology counterparts. Both opera-ms and SPAdes assemble short reads first and then improve the assembly using long reads. An alternative approach is to begin with a long-read assembly and polish the result with short reads using a dedicated tool such as Pilon [38]. However, Pilon could not be run on our complex metagenome as it required about 1000 bytes of RAM for each nucleotide in the sequence to be polished. Therefore, we mapped Illumina PE reads to the long-read assembly with Bowtie2 and created a consensus sequence with bcftools. Since canu outperformed the other long-read assemblers (Figs 2 and S4–S7, Table S3), its output was used for this polishing step, resulting in a canu+assembly approach.

The average genome fraction (94.3 %), fragmentation (16.7) and number of chimeric contigs (2.82) were best for the canu+approach with ONT reads (Figs 2 and S4–S7, Table S3). These results are comparable with other studies indicating that polished canu assemblies were the best hybrid approach [12,22]. However, SPAdes and opera-ms with Illumina PE and PacBio HiFi reads outperformed the canu+approach with PacBio HiFi reads using these metrics, likely because of the higher effective coverage provided by the Illumina PE reads. In terms of error rate, SPAdes (0.27 %) outperformed opera-ms (0.30 %), but not canu+ (0.15 %) (Figs 2 and S4, Table S3). Compared to single-technology approaches, the hybrid assembly with ONT reads by SPAdes and opera-ms performed worse than assemblers relying solely on ONT reads, except for error rate (Figs 2 and S4–S7, Table S3). In contrast, canu+outperformed canu by decreasing the high error rate, which was the main drawback of assemblies based solely on ONT reads. Therefore, we conclude that the use of short reads to perform error correction on ONT read assemblies is preferred above using them to build the assemblies, unless error rate is critical. These findings are in line with other studies [12,13, 22]. The benefit of hybrid assembly with PacBio HiFi reads was less evident. Compared with the canu PacBio HiFi assembly, canu+did not strongly affect error rate (0.18 % versus 0.15 %), and while SPAdes and opera-ms with Illumina PE and PacBio HiFi reads increased genome fractions (from 61.5–83.2 % and 81.7 %), they also increased fragmentation (from 167 to 425 and 635) and error rate (from 0.18–0.27 % and 0.30 %). These limitations could be bypassed by increasing the coverage of our HiFi reads at least three times, in order to achieve an effective coverage comparable with the lowest ONT coverage. However, in highly complex metagenomes, where such a high coverage is sometimes unfeasible, the low error rate of pure HiFi assemblies probably does not warrant the addition of Illumina PE reads if accuracy is important in addressing the research question.

Overall, when the coverage by long reads is sufficient, short reads should be primarily used for polishing. However, if coverage by long reads is not sufficient to reconstruct high genome fractions, as was the case with our PacBio HiFi reads, SPAdes should be the preferred assembler as it performed better than opera-ms when all PacBio HiFi reads were used.

### Performance with subsampled data

The datasets used in this study had a rather deep coverage of around 74- to 82-fold, though the PacBio HiFi reads had only an effective coverage of around sevenfold. Nevertheless, such sequencing depth may only be reached for the highly abundant strains in metagenomes with a high differential strain abundance. Therefore, we subsampled the read datasets, simulating high- (~70×), medium- (~35×), and low-coverage (~20×) situations (Fig. 1), in order to evaluate how assemblers perform as sequencing depth decreases, i.e. simulating cases where community members cannot be sequenced as deeply or projects where samples are pooled. Two variants of subsampling were used for the hybrid approaches. In a first approach, the complete Illumina PE dataset was used with different long-read fractions, which would simulate a case where Illumina PE sequencing is the cost-efficient method for generating the bulk output. In a second approach, short and long reads were simply subsampled to an equal depth. Noteworthy, since the actual output of the PacBio sequencer consists of subreads, these were used as the basis for subsampling rather than HiFi reads.

#### Single-technology assemblies

**Fig. 5.**
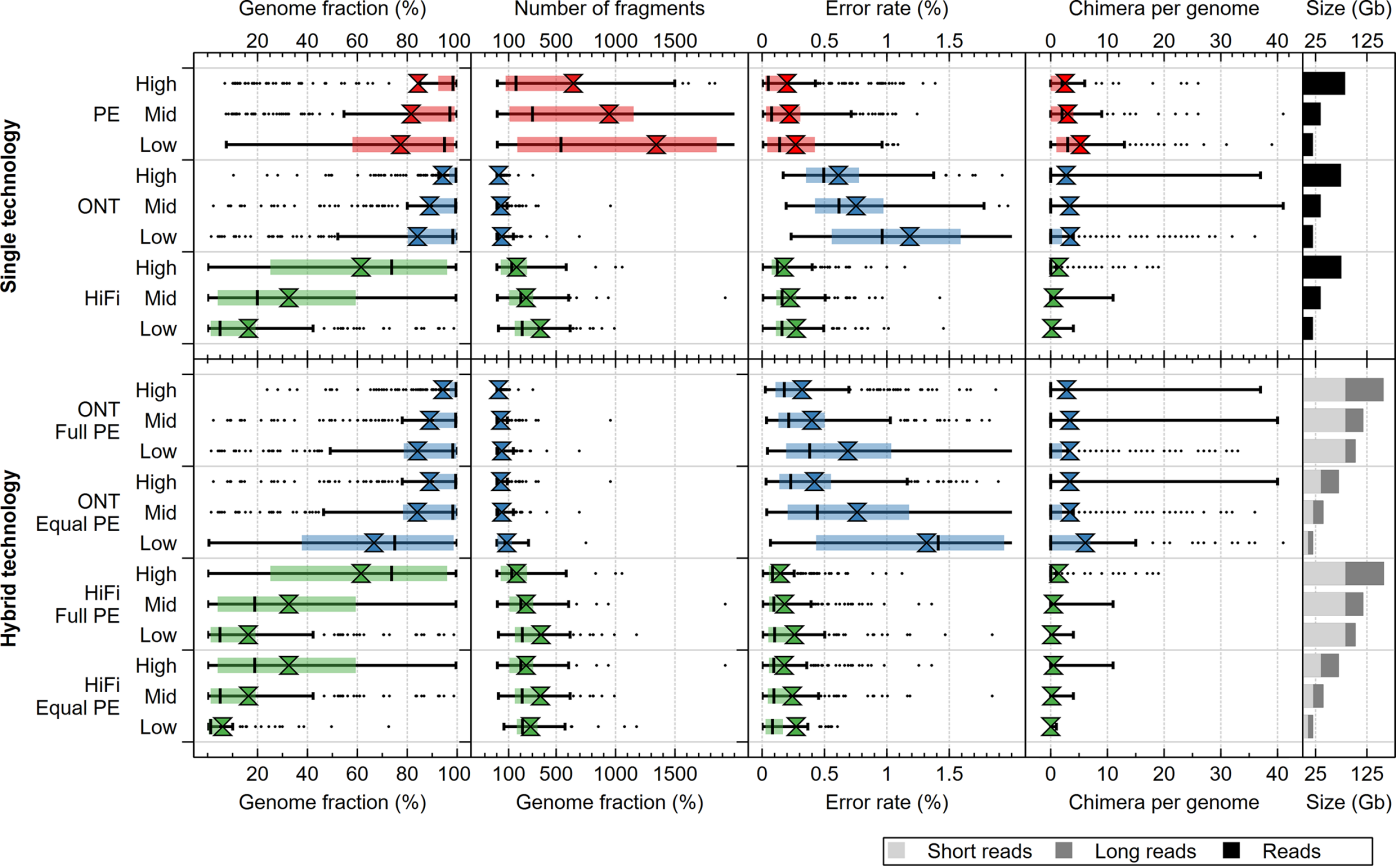
Box plots with the 1.5 IQR (interquartile range) of genome fraction, fragmentation, error rate and inter-reference misassemblies (chimaeras) collected for SPAdes (single technology) and canu+ (hybrid assembly) on subsampled datasets. Assemblers were evaluated according to four criteria: genome fraction (i.e. the fraction of the reference genome that was found it the assembled metagenome), genome fragmentation (i.e. the number of fragments, computed by dividing the number of contigs by the genome fraction), error rate (i.e. the sum of the number of mismatches and the length of all short indels), and the number of inter-reference misassemblies or chimaeras (i.e. the number of contigs that partially mapped to another genome). The size of the input dataset is shown on the right.

For Illumina PE assemblies, SPAdes performed best overall. The assemblies based on lower coverage had smaller genome fractions, higher fragmentation rates, increased error rates and increased number of inter-reference misassemblies (chimaeras) (Figs 5 and S4–S7, Table S3). However, this decrease in performance was not proportional to the decrease in coverage. For example, genome fraction dropped from 83.4 % (high coverage) to 77.4 % (low coverage) despite a drop in coverage from above 80- to 20-fold, respectively. The primarily affected metric was fragmentation, which was also the main weakness of the Illumina PE assemblies. megahit showed a more stable performance in terms of error rates and had a slightly higher genome fraction than SPAdes (Table S3). Nevertheless, the higher genome fragmentation and very high number of chimeric contigs made megahit less useful than SPAdes for reconstructing genomes irrespective of coverage, and for analysis of individual genes when coverage was sufficiently high. However, when coverage was very low, megahit outperformed SPAdes for reconstructing individual genes. We checked this by counting for each genome the number and ANI of complete genes in the final assembly versus the reference assembly (Table 2). Despite the roughly equal overall error rate for SPAdes and megahit, the error rate for complete genes (based on ANI) observed for the megahit assemblies was systematically lower than for SPAdes, when analysed per genome. This was even noticeable at higher coverage, where we expected SPAdes to perform better because of its lower overall error rate. Conversely, SPAdes recovered more complete genes per genome. This analysis showed that the choice of using megahit or SPAdes could be directed by the envisaged downstream analyses.

**Table 2.** The number of genomes in the HC227 mock for which one assembler outcompetes the other based on the count or ANI of complete genes recovered from the assemblies

Metric	Assembler	Dataset		
		Full	35 Gb	20 Gb
Gene count	SPAdes	149	149	153
		78	78	74
ANI	SPAdes	45	47	60
		182	180	167

For ONT assemblies, canu had an overall better performance than Flye. As coverage decreased, the primarily affected metrics where error rate and genome fragmentation, which almost doubled and tripled, respectively (Figs 5 and S4–S7, Table S3). Nevertheless, fragmentation remained much lower than with other technologies. With the low-depth dataset, Flye slightly outperformed canu on completeness (85.0 % versus 84.1 %) and error rate (0.89 % versus 1.18 %), suggesting that this assembler might be more suited for assembling genomes when coverage is below 20-fold, as the performance of Flye degraded less than that of canu with decreasing coverage.

For PacBio HiFi reads assembled with Flye and canu, the genome fraction decreased proportionally to the amount of data, which was a consequence of the low effective coverage (Figs S4–S7, Table S3). canu outperformed Flye for all metrics except for the average genome fraction. Although another study showed that Flye had a more robust performance on subsampled datasets [54], this is not contradictory since we subsampled on grouped subreads rather than by randomly selecting subreads.

For long reads in general, we found that Flye produced more contiguous assemblies than canu if the overall coverage was low (≤20×). The number of genome fragments produced by canu decreased with increasing coverage, whereas assemblies produced by Flye seemed to stagnate (around 45 genome fragments for ONT reads). These results are in agreement with the observation that Flye produced larger assemblies than canu for 3 GB of sequencing data of real metagenomes [22] and for low-abundant genomes in commercially available mocks [12]. As such, we conclude that the advantage of canu can only be observed if sequencing is sufficiently deep. However, one major advantage that Flye had over canu at high coverage is the required run time (Table 3), which was about two orders of magnitude lower than canu. In our case, processing the full ONT data (74.6 Gb) with canu required over 21 days, running on 54 cores (Table 3).

**Table 3.** User time (h) required for assemblers depending on input data size

Method	Assembler	Dataset		
		Full	35 Gb	20 Gb
Illumina	SPAdes	720.5	321.2	238.0
		313.0	177.3	126.0
Nanopore	CANU	39 249.4	10 796.2	5232.2
	Flye	212.7	101.0	60.2
PacBio (CCS)	CANU	96.9	22.9	8.2
	Flye	22.3	8.8	4.6
ONT +Full PE	SPAdes	939.9	814.5	849.8
	OPERA-MS	427.9	430.2	357.7
ONT +Equal PE	SPAdes	490.2	297.9	185.0
	OPERA-MS	278.1	171.5	107.0
HiFi+Full PE	SPAdes	830.4	825.1	861.6
	OPERA-MS	348.1	339.1	291.6
HiFi+Equal PE	SPAdes	382.8	233.2	168.7
		136.0	90.4	57.5

#### Hybrid assemblies

For the Illumina PE-ONT assemblies, although the canu+approach still outperformed all other methods (Figs S6 and S7, Table S3), the assembly completeness decreased faster with decreasing ONT coverage than for the other hybrid assemblers. This was expected because SPAdes and opera-ms do not rely on ONT reads for the construction of contigs. In addition, the error rates of both SPAdes and opera-ms increased slower for both subsampling approaches. For completeness, the best-performing tool was opera-ms, except for the full dataset, where it was outperformed by canu+. Interestingly, unlike all other assemblers, the average number of chimeric contigs for opera-ms decreased from 19.37 to 16.61 and 14.23 with decreasing coverage of ONT reads for the datasets with the full Illumina PE data, whereas an increase could be observed for all other approaches (Figs S5 and S6, Table S3). However, it is important to note that these values were considerably lower for the other approaches (Table 3). For the equal-depths datasets, this trend was not observed, as the average number of chimeric contigs increased as the coverage decreased.

Finally, for the Illumina PE-HiFi assemblies, the canu+approach did not produce a sufficiently large genome fraction to be useful (with the exception of the full dataset, the genome fraction was <40 % in all cases). Noteworthy, opera-ms had a more stable performance than SPAdes. Its error rate was lower than all other approaches for both low datasets, and the equal PE-HiFi dataset. Unlike the other approaches, this error rate also decreased slightly as depth decreased (both for the full-PE and equal-depths cases), making it the best approach for hybrid assembly of PE-PacBio reads when coverage is low.

Overall, the canu+approach, while effective when coverage was high, seemed to degrade rapidly with decreasing coverage. Therefore, it should not be used when coverage is low. Combined with long computational times (Table 3), this approach is likely impractical in many cases. The alternatives considered here – SPAdes and opera-ms – produced less contiguous assemblies with more errors, but were able to reconstruct more of the input genomes when coverage was low. For PE-ONT assemblies, opera-ms produced a larger genome fraction with lower fragmentation, but had higher error rates, regardless of the coverage, whereas this trend seems to be reversed for PE-HiFi assemblies. Depending on the weight of the evaluation criteria for a given application, either assembler may be more suitable.

We conclude that with sufficient coverage, assemblies should be constructed using long reads and polished using short reads, rather than using long reads to construct scaffolds for short-read assemblies. Furthermore, we hypothesize that the coverage of ONT reads needs to be between half and equal the Illumina PE coverage in order to improve genome fractions with the canu+approach (Table S3).

## Discussion

In this study, we evaluated the performance of different sequencing and assembly approaches on a complex mock community. Our results indicated that for the same amount of sequencing data, ONT sequencing was most effective for reconstructing complete genomes. Nevertheless, our ONT sequencing data still had an error rate above 10 %, which resulted in high error rates in the assembly. Noteworthy, ONT reads showed higher error rates as this study was performed with R9.4.1 chemistry. With the more recently released R10.4 chemistry, read accuracy is comparable to that of Illumina reads [56], which probably will also improve assembly. As a result, we conclude that metagenomes produced with ONT likely contain too many errors to be used for detecting variants. If the latter is the main goal, PacBio HiFi sequencing would be preferred over ONT sequencing, but this comes at a cost as coverage would need to be increased significantly to obtain similar genome fractions (for our dataset from 70 to 100 Gb). Related to coverage, HC227 contains even amounts of DNA for each of the 227 strains. This was done in part to increase the difficulty of binning the mock correctly for benchmarking other aspects of the metagenome-assembled genome workflow [28]. However, real microbial communities are not evenly distributed, which also will affect the coverage needed (depending on the research question). Noteworthy, PacBio HiFi-based assemblies were more prone to indels than Illumina PE, as such we expect that (predicted) protein sequences are more likely to contain errors. Although hybrid assembly of Illumina PE and ONT reads could reduce the error rate, this improvement is probably not enough to warrant a hybrid approach. The ability of assemblies based solely on long reads is sufficient to resolve significant fractions of genomes as single sequences, and the error rate of assemblies based on short reads is better than that of the hybrid one. Similarly, hybrid assembly of Illumina PE and PacBio HiFi reads did not significantly improve the results when compared to the corresponding single-technology approaches, especially considering that the accuracy with PacBio HiFi reads is much closer to that of Illumina PE reads than ONT reads. Therefore, with the current assemblers, Illumina PE sequencing and assembly remains superior to long-read as well as hybrid assembly for complex metagenomes in cases where a low error rate is critical. However, the fragmented nature of these assemblies makes it difficult to separate it into component genomes, and if that is the aim of a study, an assembly based on only long reads is more appropriate. Although short reads may be useful to polish such long-read assemblies, this produces only a marginal improvement with currently available software. Although not directly achievable with the current tools, one could expect that if a hybrid approach could efficiently extract and combine the best results of the short- and long-read single-technology assemblies, hybrid assembly would be the approach of choice and yield a contiguous and complete assembly with a low error rate.

It became evident that many of the tested assemblers are not ready to assemble complex metagenomes on moderately powerful computing infrastructure. Of the assemblers initially considered, only SPAdes, Flye, CANU, OPERA-MS and WTDBG could be run successfully on all datasets. Moreover, the assemblers that were designed to handle hybrid metagenome have clear flaws. They produced worse completeness and higher fragmentation than their long-read counterparts and were more likely to assemble contigs incorrectly. Until these flaws are corrected, long-read assembly followed by a polishing step using Illumina reads is likely to yield better results for ONT sequencing.

In essence, our work can be used as a guide to select the correct sequencing technology (and assembler) depending on the specific goals, the complexity of the genome(s) or region(s) of interest, desired accuracy, budget, computational capabilities and throughput requirements. In addition, our work showcases issues with the present assemblers that need to be addressed to improve the quality of bacterial metagenomes.

## supplementary material

10.1099/mic.0.001469 Table S1.

10.1099/mic.0.001469 Table S2.

10.1099/mic.0.001469 Table S3.

10.1099/mic.0.001469 Fig. S1.

10.1099/mic.0.001469 Fig. S2.

10.1099/mic.0.001469 Fig. S3.

10.1099/mic.0.001469 Fig. S4.

10.1099/mic.0.001469 Fig. S5.

10.1099/mic.0.001469 Fig. S6.

10.1099/mic.0.001469 Fig. S7.

10.1099/mic.0.001469 Fig. S8.

## Abbreviations

ANI: average nucleotide identity
bp: base pair
CCS: circular consensus sequencing
ENA: European Nucleotide Archive
Gb: Gigabase
HiFi: high-fidelity
IQR: interquartile range
ONT: Oxford Nanopore Technologies
PacBio: Pacific Biosciences
PE: paired-end
ZMW: zero-mode waveguide
