# Supplementary material for: Benchmarking short-, long- and hybrid-read assemblers for metagenome sequencing of complex microbial communities
Source: Microbiology (Reading). 2024 Jun 25;170(6):001469. doi: 10.1099/mic.0.001469 (PMC11261854; doi:10.1099/mic.0.001469)
Supplement: Fig. S1. [file mic-170-01469-s004.pdf]

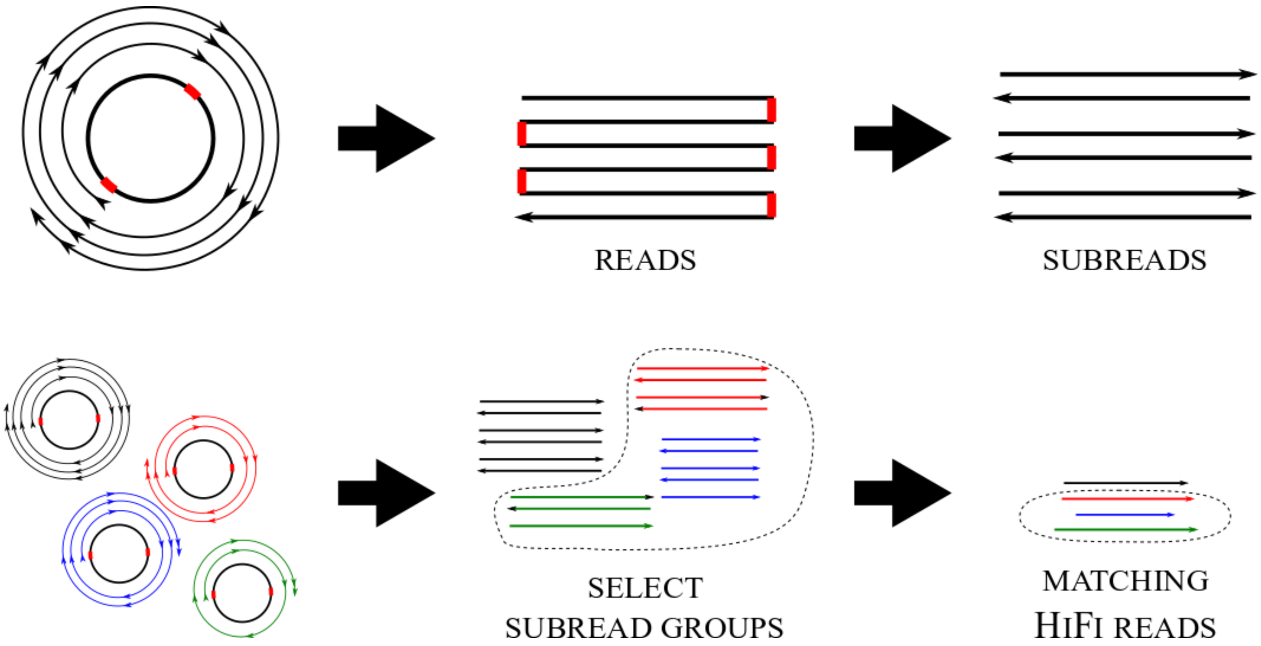

Supplementary Figure 1. Procedure used to subsample PacBio HiFi reads. PacBio sequencing creates a continuous string which represents multiple passes around the same circularized double-stranded DNA fragment. This continuous string is split into a group of subreads when the circularizing adapters are removed. PacBio HiFi reads were generated for all subread groups using the PacBio CCS tool. When subsampling, groups of subreads were randomly selected up to a specified total length, and only those that were generated from one of the selected subread groups were kept.
