# Supplementary material for: Benchmarking short-, long- and hybrid-read assemblers for metagenome sequencing of complex microbial communities
Source: Microbiology (Reading). 2024 Jun 25;170(6):001469. doi: 10.1099/mic.0.001469 (PMC11261854; doi:10.1099/mic.0.001469)
Supplement: Fig. S2. [file mic-170-01469-s005.pdf]

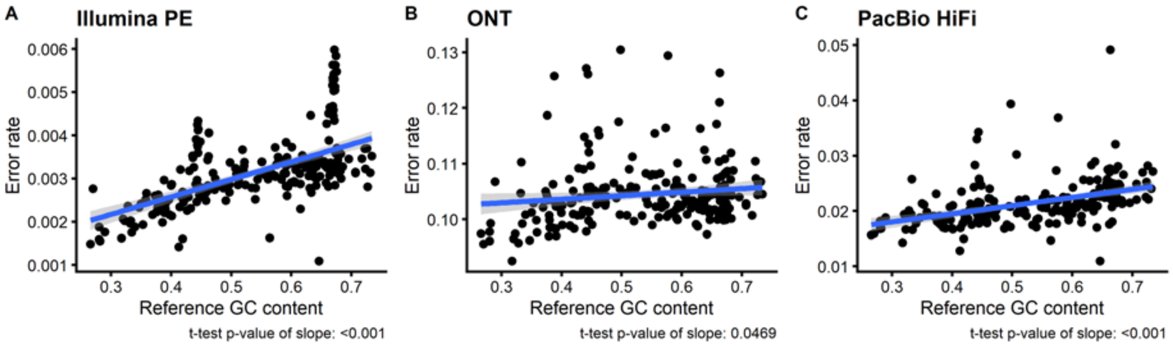

Supplementary Figure 2. Per-genome error rates according to GC content of the reference. Illumina PE (A), ONT (B) and PacBio HiFi (C) reads were aligned using minimap2 and grouped according to the genome from which they originated. The error rate for each genome was then computed as the sum of single nucleotide mismatches, the total length of insertions and the total length of deletions. A linear regression model was fitted using the lm() function in R and the output called with the summary() function showed if the slope coefficient was statistically significant.
