# Supplementary material for: Benchmarking short-, long- and hybrid-read assemblers for metagenome sequencing of complex microbial communities
Source: Microbiology (Reading). 2024 Jun 25;170(6):001469. doi: 10.1099/mic.0.001469 (PMC11261854; doi:10.1099/mic.0.001469)
Supplement: Fig. S3. [file mic-170-01469-s006.pdf]

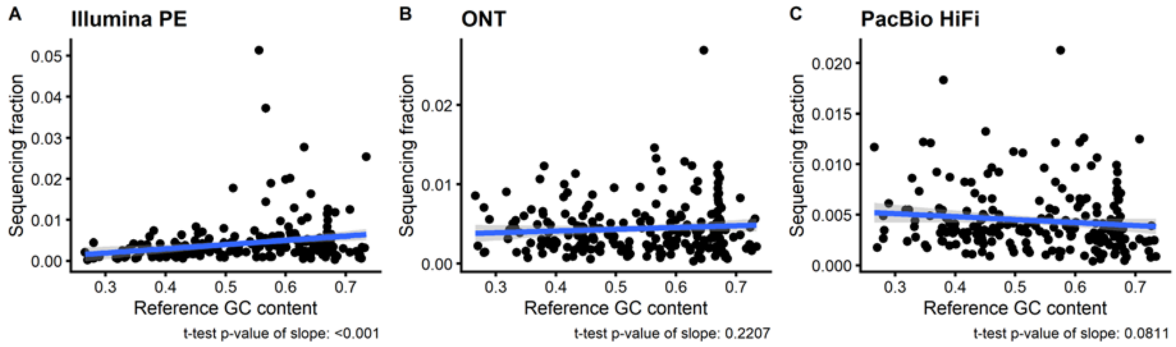

Supplementary Figure 3. Per-genome fraction of sequencing data according to GC content of the reference. Illumina PE (A), ONT (B) and PacBio HiFi (C) reads were aligned using minimap2 and grouped according to the genome from which they originated. The lengths of all reads in each group were summed up and divided by the sum of the length of all reads to compute the sequenced fraction of each genome. A linear regression model was fitted using the lm() function in R and the output called with the summary() function showed if the slope coefficient was statistically significant.
