# Supplementary material for: Benchmarking short-, long- and hybrid-read assemblers for metagenome sequencing of complex microbial communities
Source: Microbiology (Reading). 2024 Jun 25;170(6):001469. doi: 10.1099/mic.0.001469 (PMC11261854; doi:10.1099/mic.0.001469)
Supplement: Fig. S4. [file mic-170-01469-s007.pdf]

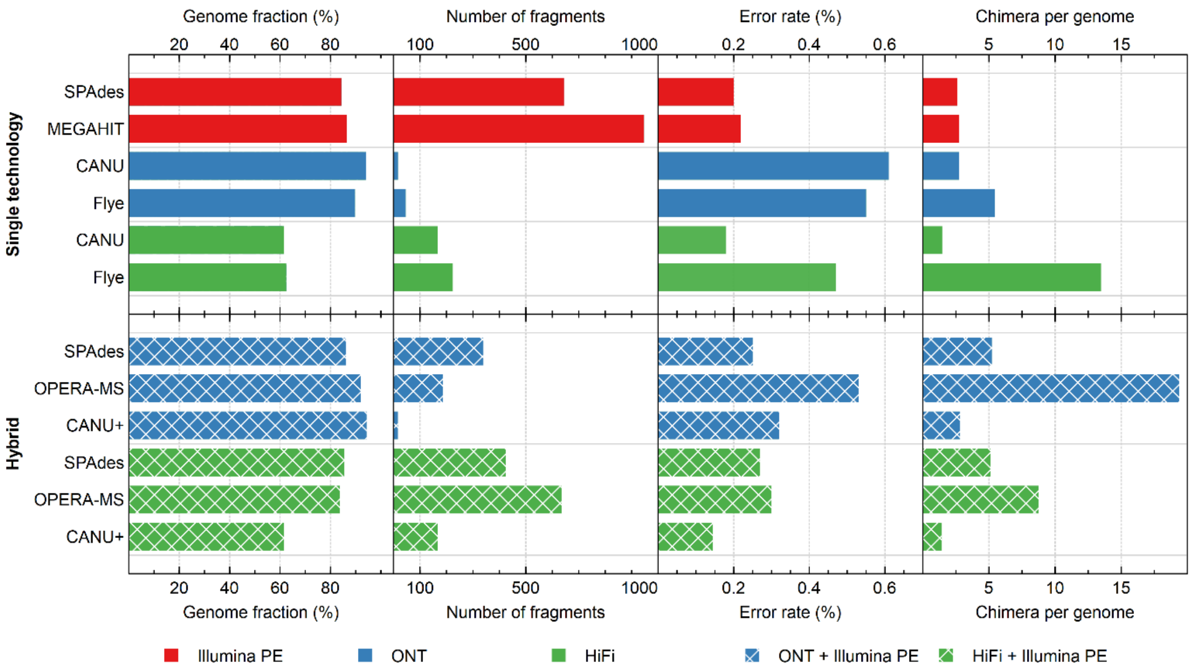

Supplementary Figure 4. General assembly performance of each tool on single-technology and hybrid datasets. Genome fraction, fragmentation and error rate were collected for each reference and **averaged to produce a single value**. Fragmentation was computed by dividing the number of contigs by the genome fraction.
