# Supplementary material for: Benchmarking short-, long- and hybrid-read assemblers for metagenome sequencing of complex microbial communities
Source: Microbiology (Reading). 2024 Jun 25;170(6):001469. doi: 10.1099/mic.0.001469 (PMC11261854; doi:10.1099/mic.0.001469)
Supplement: Fig. S5. [file mic-170-01469-s008.pdf]

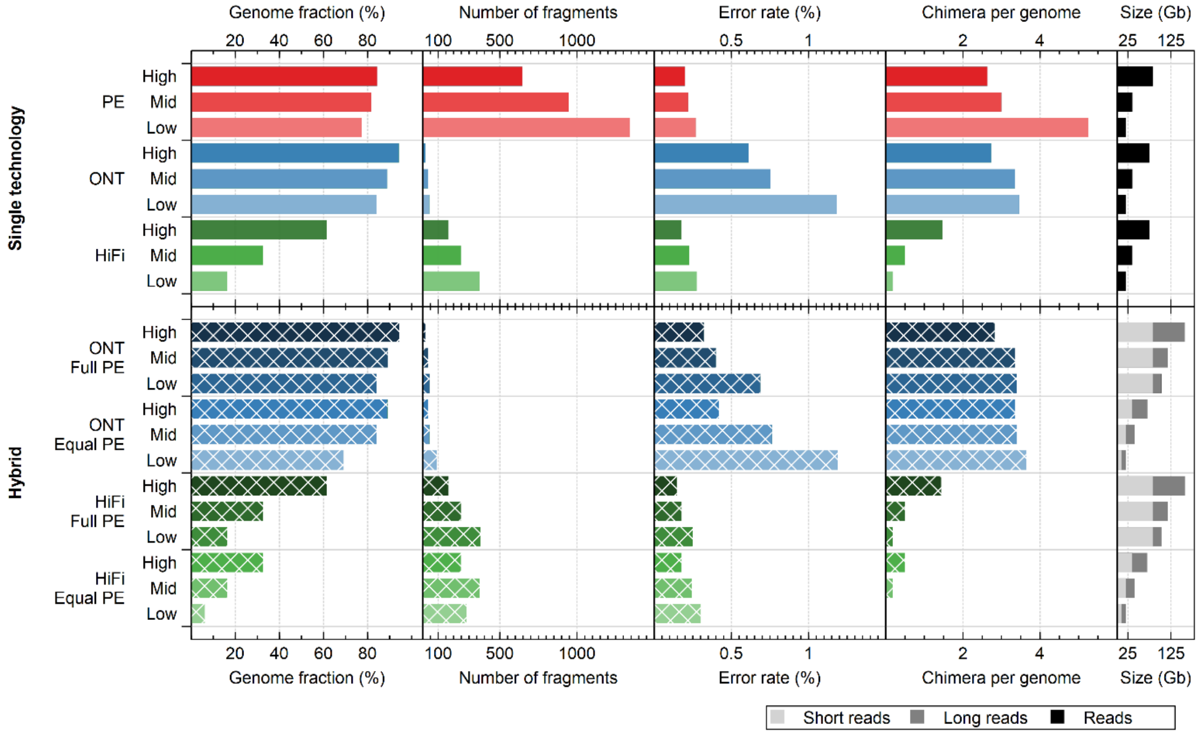

Supplementary Figure 5. General assembly performance of SPAdes (single technology) and CANU+ (hybrid assembly) on subsampled datasets (sizes are indicated in far right column). Genome fraction, fragmentation and error rate were collected for each reference and **__averaged to produce a single value__**. Fragmentation was computed by dividing the number of contigs by the genome fraction.
