# Supplementary material for: Benchmarking short-, long- and hybrid-read assemblers for metagenome sequencing of complex microbial communities
Source: Microbiology (Reading). 2024 Jun 25;170(6):001469. doi: 10.1099/mic.0.001469 (PMC11261854; doi:10.1099/mic.0.001469)
Supplement: Fig. S6. [file mic-170-01469-s009.pdf]

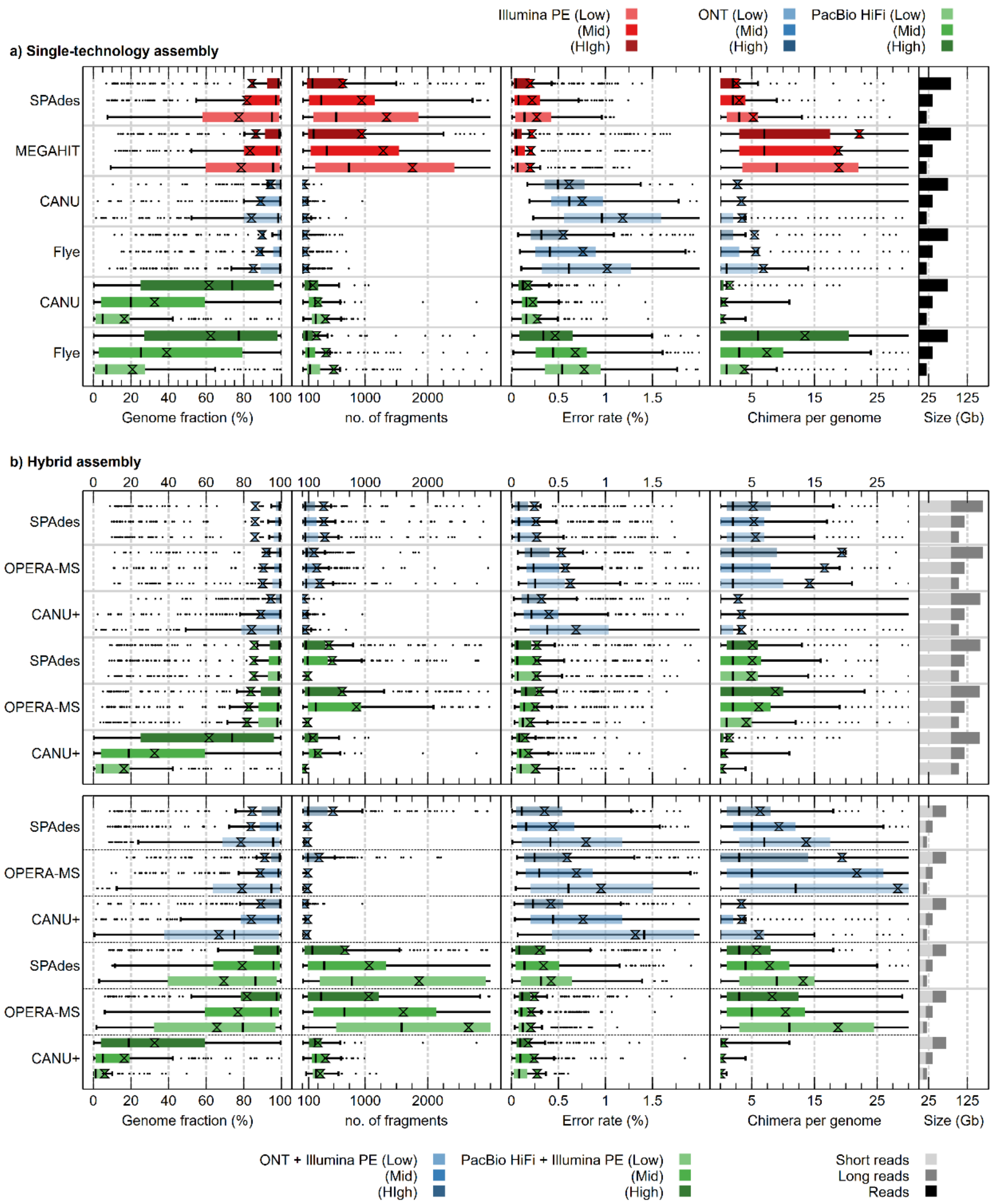

Supplementary Figure 6. Box plots with the 1.5 IQR (Interquartile range) of genome fraction, fragmentation, error rate and interspecies translocations collected for the different assemblers on the full and subsampled datasets for single (a) and hybrid (b) technology datasets. Assemblers were evaluated according to four criteria: genome fraction (i.e. the fraction of the reference genome that was found it the assembled metagenome), genome fragmentation (i.e. the number of fragments, computed by dividing the number of contigs by the genome fraction), error rate (i.e. the sum of the number of mismatches and the length of all short indels), and the number of inter-reference misassemblies or chimeras (i.e. the number of contigs that partially mapped to another genome). The size of the input dataset is shown on the right. Hybrid assemblies were performed with either full Illumina PE reads and subsampled long reads (b, top panel) and equally subsampled short and long reads (b, bottom panel).
