# Supplementary material for: Benchmarking short-, long- and hybrid-read assemblers for metagenome sequencing of complex microbial communities
Source: Microbiology (Reading). 2024 Jun 25;170(6):001469. doi: 10.1099/mic.0.001469 (PMC11261854; doi:10.1099/mic.0.001469)
Supplement: Fig. S7. [file mic-170-01469-s010.pdf]

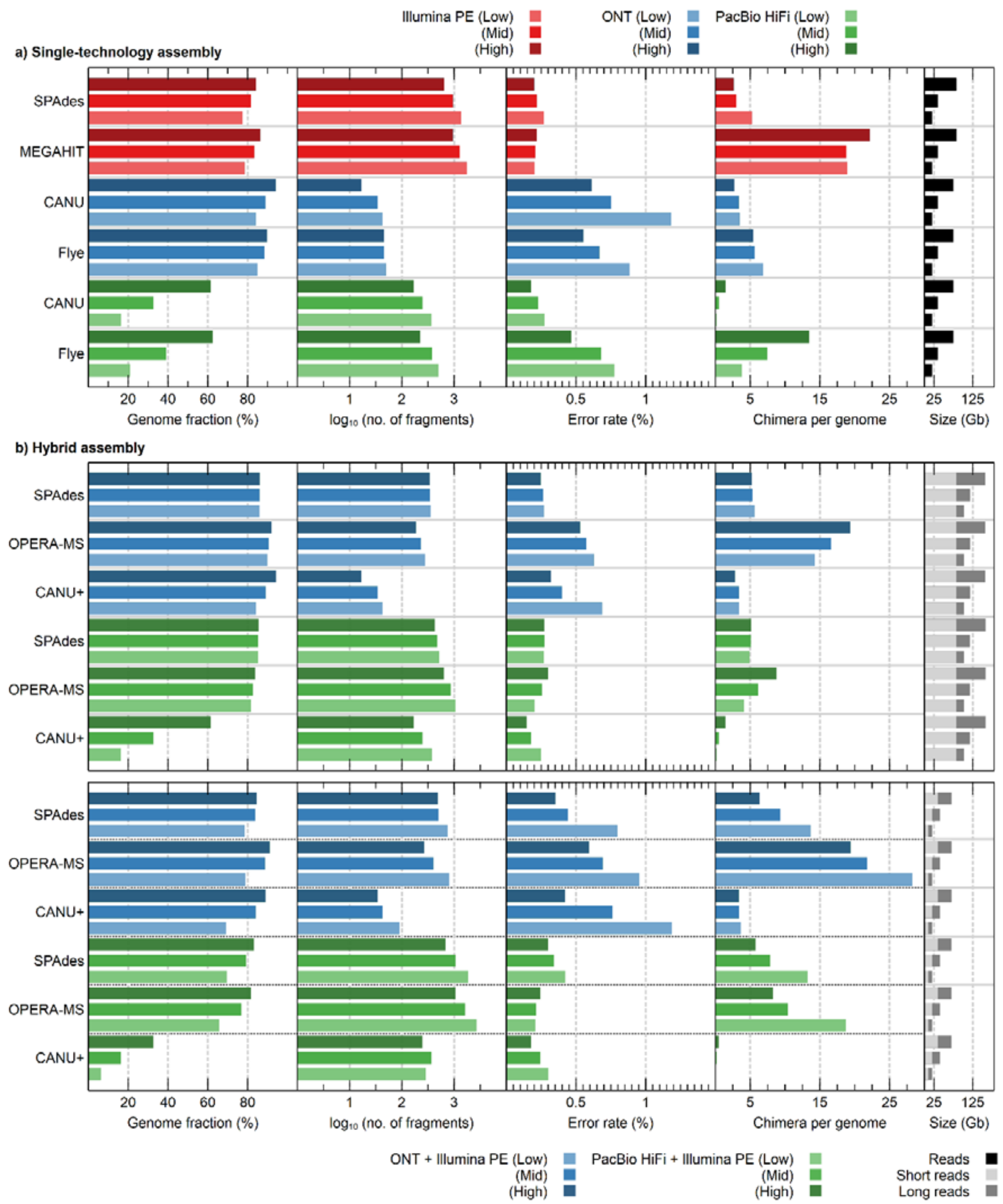

Supplementary Figure 7. Performance summary of assemblers for the full and subsampled datasets for single-technology (a) and hybrid assemblies (b). Assemblers were evaluated according to four criteria: genome fraction (the **average** fraction of the reference genome that was found it the assembled metagenome), genome fragmentation (the **average** number of contigs), error rate (the **average** of the sum of the number of mismatches and the length of all short indels), and the number of chimeras (the **average** number of contigs that partially mapped to another genome). The size of the input dataset is shown on the right. Hybrid assemblies were performed with either full Illumina PE reads and subsampled long reads (b, top panel) and equally subsampled short and long reads (b, bottom panel).
