# Supplementary material for: Benchmarking short-, long- and hybrid-read assemblers for metagenome sequencing of complex microbial communities
Source: Microbiology (Reading). 2024 Jun 25;170(6):001469. doi: 10.1099/mic.0.001469 (PMC11261854; doi:10.1099/mic.0.001469)
Supplement: Fig. S8. [file mic-170-01469-s011.pdf]

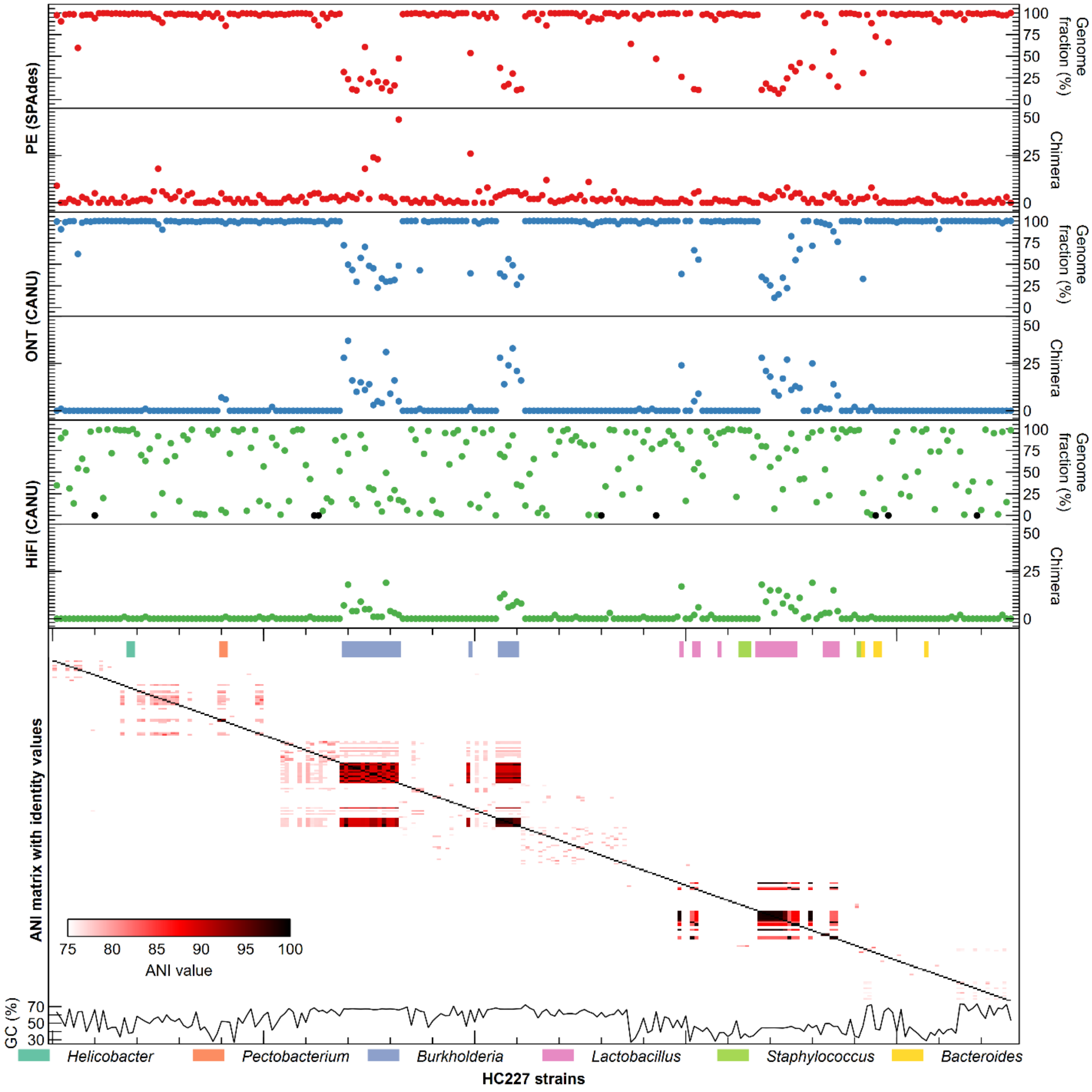

Supplementary Figure 8. Genome fractions and the number of chimeras (the average number of contigs that partially mapped to another genome) of the different strains in HC227 assembled by SPAdes starting from Illumina PE reads (red), and CANU starting from ONT (blue) and HiFi (green) reads (black represents genomes that were not assembled). Genomes are ranked based on the ANI matrix with identity values, genomes from the same genus are color-coded (above ANI matrix). GC content of the genomes is shown below the ANI matrix.
